# A case report of hemorrhagic presentation of diffuse neonatal hemangiomatosis (DNH) associated to obstructive hydrocephalus: Medical and neurosurgical considerations

**DOI:** 10.1007/s00381-023-06226-2

**Published:** 2023-11-18

**Authors:** Marco Pavanello, Liliana Piro, Arianna Roggero, Andrea Rossi, Gianluca Piatelli, Nadia Vercellino

**Affiliations:** 1 Department of Neurosurgery, IRCCS Istituto Giannina Gaslini, Via G.Gaslini, Genoa, Italy; 2 Pediatric Surgery Unit, IRCCS Istituto Giannina Gaslini, Via Gerolamo Gaslini 5, 16147 Genoa, Italy; 3 University od Genoa, DINOGMI, Genoa, Italy; 4 Neuroradiology Unit, IRCCS Istituto Giannina Gaslini, Via G.Gaslini, Genoa, Italy; 5 Department of Health Sciences (DISSAL), University of Genoa, Genoa, Italy; 6 Cardiovascular Department, IRCCS Istituto Giannina Gaslini, Via G.Gaslini, Genoa, Italy

**Keywords:** Diffuse neonatal hemangiomatosis, Ventriculocisternostomy, Obstructive hydrocephalus, Case report

## Abstract

**Background:**

Diffuse neonatal hemangiomatosis (DNH) is a rare disorder typically recognized at birth or during the neonatal period. DNH involves three or more organ systems, including the central nervous system (CNS). In these cases, serious complications such as hemorrhages and obstructive hydrocephalus can develop.

**Case report:**

We present a case of DNH with intracranial hypertension and CNS hemorrhagic lesions on the mesencephalic aqueduct, resulting in triventricular hydrocephalus, treated with endoscopic ventriculocisternostomy (ETV) and medical therapy.

**Discussion:**

DNH is a rare condition that can involve the CNS with serious complications. From a review of the literature, we highlighted only two cases of DNH with brain involvement treated surgically. We report the successful outcome of ETV, along with surgical considerations, imaging findings, and the complete resolution of cerebral and skin lesions following medical therapy.

**Conclusions:**

Medical therapy is not standardized and must be individualized. In patients who develop severe neurological symptoms such as obstructive hydrocephalus, surgery may be considered to avoid neurological sequelae.

## Introduction

Diffuse neonatal hemangiomatosis (DNH) is a rare and often fatal condition that presents during the neonatal period [1]. It is characterized by numerous cutaneous and visceral hemangiomas with no evidence of malignancy, involving three or more organ systems, including the central nervous system (CNS) [1, 2]. We present a case of DNH with multiple cutaneous hemangiomas and the onset of cerebral hemorrhagic lesions and triventricular hydrocephalus. We describe the successful neurosurgical and medical combination therapy with a favorable outcome.

## Patient presentation

**Fig. 1:**
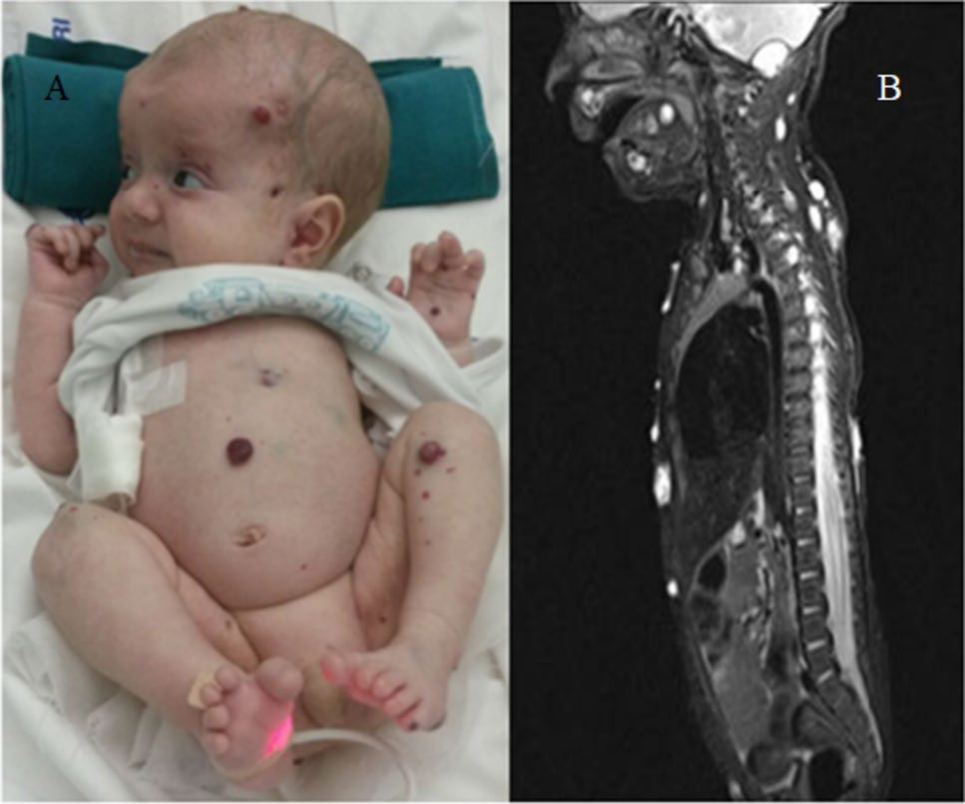
**A** Multifocal cutaneous red skin lesions of the face, trunk, abdomen, and limbs; **B** sagittal T2-weighted images (T2WI) showing subcutenous and subfascial lesions

**Fig. 2:**
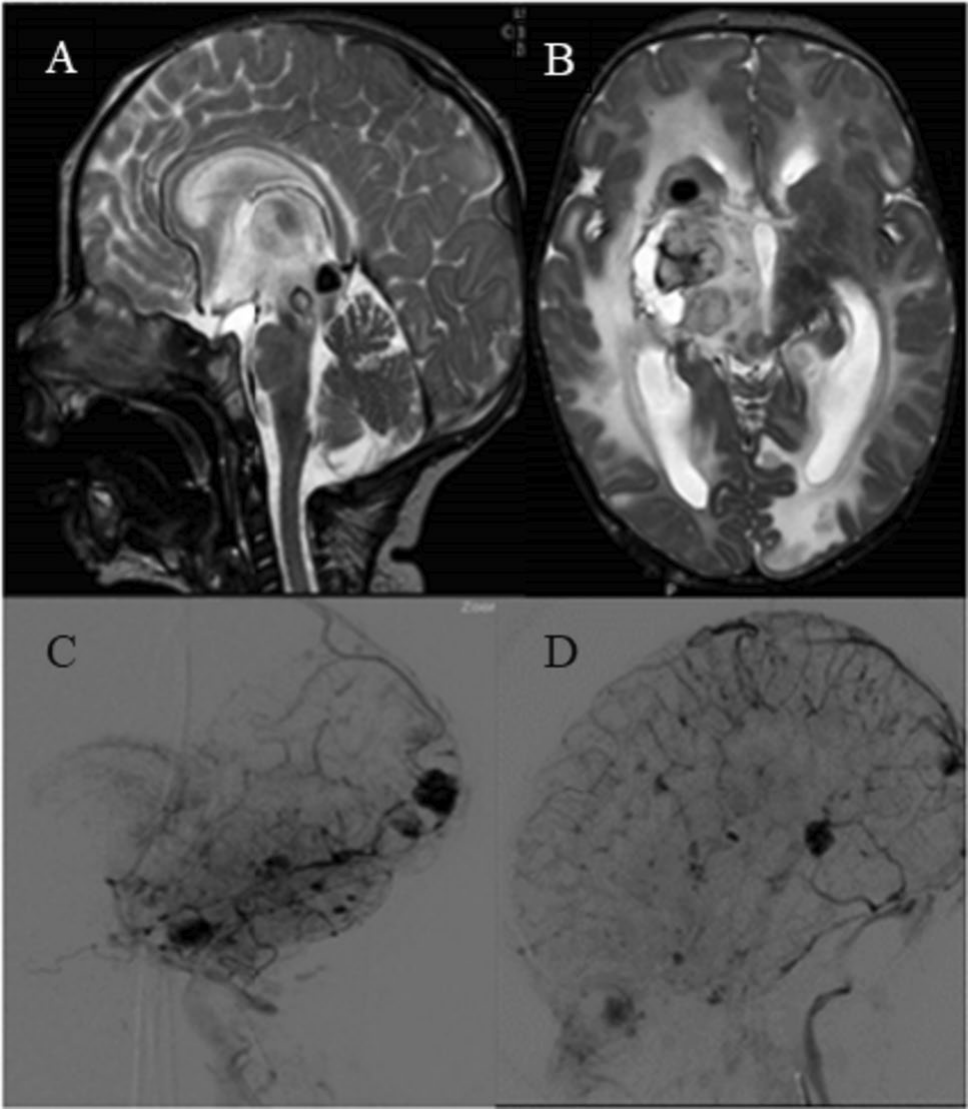
**A**, **B** At initial presentation in sagittal and axial T2-weighted images (T2WI) showing acute and multiple (nucleo-capsular right and tectal mesencephalic) ICH (intracerebral hemorrhages) with periventricular brain vasogenic edema of white matter. **C**, **D** DSA venous phase excluded arteriovenous malformation (AVM) and arteriovenous fistulas (AVFs)

**Fig. 3:**
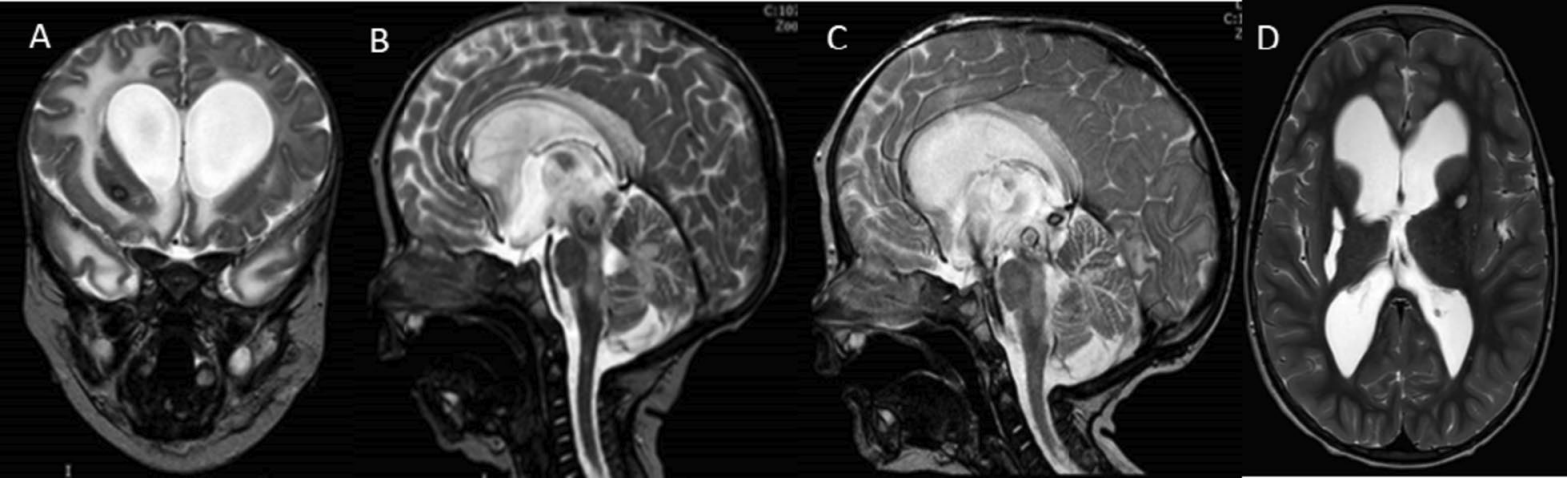
**A**, **B** After 48 h of diagnosis: coronal and sagittal T2-weighted images (T2WI) showed a tectal mesencephalic hemorrhage with obstructive hydrocephalus due to aqueductal stenosis associated enlargement of the recesses of the third ventricle as finding of increased intracranial pressure. **C** After 24 h of surgery, a sagittal T2WI showed the cerebrospinal fluid flow artefact in the third ventricle sign of successful ETV. **D** After 10 years, a coronal MRI (T2WI) showed residual lacunar sequelae right nucleo-capsual and the regression of vascular lesions

A 9-week-old male infant presented multiple diffuse red skin lesions. These swellings gradually increased in number and size and were associated with subcutaneous harder blue lesions (Fig. 1). Neurological examination revealed progressive macrocrania associated with irritability and vomiting. A total body angio-MR study showed multiple diffuse vascular lesions in the brain, spinal cord, bones, muscles, and viscera. Brain lesions were disseminated, involving both supra- and infratentorial regions, with intense and homogeneous contrast enhancement. The largest lesions were deep within the cerebral and cerebellar hemispheres, particularly the one located in the right lenticular nucleus, which showed acute hemorrhage with vasogenic edema, leading to compression of the mesencephalic aqueduct and resulting in triventricular hydrocephalus (Fig. 2). An endoscopic ventriculocisternostomy (ETV) was performed on the same day. Surgical approach had included a transfontanellar access on the right, with navigation references to select the entry point and define the route for the 0° Gaab rigid endoscope. Fenestration was achieved between the mammillary bodies and the infundibulum of the pituitary gland using only a Fogarty balloon. During the procedure, we noted that the ventricular ependyma had a petechial appearance, but no significant hemorrhage occurred, and only minor bleeding was controlled with continuous irrigation. A post-operative MRI showed adequate flow signal through the stoma (Fig. 3). A biopsy was performed during hospitalization to study some ulcerated and bleeding lesions, which revealed papillary endothelial hyperplasia with no pathognomonic features of a specific entity. Genetic mutations (EIF2AK4, ACVRL1, BMPR1B, BMPR2, CAV1, ENG, KCNK3, SMAD9, NOTCH3, and WES sequences) were investigated with no evidence found. Therapy was initiated with Prednisone (2 mg/kg/day) and Propranolol (1 mg/kg/day). However, due to further massive epistaxis requiring a blood transfusion and no improvement, the therapeutic scheme was modified to combine Prednisone and Vincristine. After 3 weeks of therapy with no improvement, Vincristine was replaced by Rapamycin (0.8 ml/day) with ethical committee consent. The therapy was effective, with no more bleeding observed, and the lesions gradually became smaller and clearer with some desquamative features. The patient was discharged, and therapy continued at home, with Rapamycin doses adjusted based on blood levels, while corticosteroids were gradually decreased. Follow-up over 5 years confirmed the absence of new lesions (Fig. 3).

## Discussion

DNH is a rare but significant medical condition characterized by excessive growth and proliferation of hemangiomas [1, 2]. While the exact causes of DNH are not yet fully understood, it is believed to result from a combination of genetic and environmental factors, with some studies suggesting certain genetic predispositions may increase the risk of this condition [1]. The disease may present with various symptoms, but the presence of numerous cutaneous hemangiomas is a distinctive sign. Hemangiomas can also develop in internal organs such as the liver, brain, or intestines, leading to pain or discomfort in newborns, although sometimes this condition can be asymptomatic [2]. Rare associations of DNH with hemangioblastomas (HBLs) have been reported, contributing to the development of hydrocephalus [2, 3]. Diagnosis requires a thorough medical evaluation, including radiological imaging. Holden and Alexander proposed three minimal diagnostic criteria for DNH: onset in the neonatal period, no evidence of malignancy, and involvement of three or more organ systems [4]. In cases with extensive lesions present at birth, unusual cutaneous morphologies, or widespread internal organ involvement, a skin biopsy should be obtained for a precise histopathologic diagnosis of the vascular tumor.

Treatment for DNH depends on the severity and location of hemangiomas. In many cases, cutaneous hemangiomas may resolve spontaneously over time, making careful observation sufficient in some situations. [1] Steroids can be used to reduce the size of hemangiomas and prevent potential complications [1, 2, 5, 6]. Approximately 16% of hemangiomas do not respond to steroid therapy, and b-blockers are considered the first-line systemic therapy [1]. Storch et al. proposed several possible molecular targets for Propranolol treatment, depending on the timing of treatment [1, 7]. Vincristine has been described for the treatment of life-threatening or corticosteroid-resistant hemangiomas. Furthermore, treatment with Rapamycin has been proven effective in managing large and rapidly proliferating hemangiomas [1].

From a literature review, five cases of DNH with MRI findings of brain and/or spinal lesions were identified. Hydrocephalus was described in three cases [2, 3, 5]. Four of these cases utilized medical treatment, leading to a regression in the size and number of cutaneous and visceral hemangiomas [2, 5–7]. In two cases, a surgical approach was used for the treatment of hydrocephalus [2, 3] (Table 1).

**Table 1:** We report the main clinical and surgical data of patients affected by DNH described in literature

**Case**	**Age at diagnosis (day/y)**	**Brain**	**Spinal**	**Events after diagnosis (day)**	**Evidence of hydrocephalus after diagnosis (day)**	**Treatment**	**Therapy**	**Time follow-up (day)**	**Outcome**
**A**^**3**^	5 day	Multifocal hemorrhagic	No	37	37	Suboccipital craniotomy and decompression	No	42	Improved
**B**^**5**^	3 day	Multifocal hemorrhagic	Multifocal hemorrhagic	9	13	n/d	Prednisolone interferon	17	Improved
**C**^**6**^	90 day	Multifocal hemorrhagic	Multifocal hemorrhagic	7	No	n/d	Dexamethasone prednisone	30	Complete resolution
**D**^**2**^	5 day	Unifocal hemorrhagic	No	n/d	n/d	Total resection and ventriculoperitoneal shunting	Steroid therapy	270	Improved
**E**^**7**^	39 day	Multifocal hemorrhagic	No	21	No	n/d	Propanololo	30	Improved
**Case report**	1 day	Multifocal hemorrhagic	Multifocal hemorrhagic	30	93	ETV	Propanololo Steroid Vincristine Rapamycin	1826	Complete resolution

This table shows the age at diagnosis of DNH expressed in days and describes the brain and spinal cord involvement. We evaluated the time of onset of cerebral hemorrhagic events and the development of hydrocephalus from the diagnosis of DNH. Finally we highlighted the surgical and medical treatment used in each case and the outcome

In cases where hemangiomas cause significant problems or pose a risk to the patient’s life, surgical intervention may be necessary to remove or treat them. [1] In cases with diffuse skin lesions, invasive procedures such as tunneling for ventriculoperitoneal (VP) shunts carry a high risk of visceral or cutaneous and subcutaneous soft tissue injury. Thus, such procedures should be discouraged. Other alternatives can be temporary, such as the placement of a reservoir or an external ventricular drain. In rare cases like our patient’s, where acute obstructive hydrocephalus appeared in the presence of multiple cutaneous and visceral lesions, early endoscopic ventriculocisternostomy (ETV) can be performed.

## Conclusions

In conclusion, DNH is a rare and complex clinical condition that requires timely and appropriate treatment [1]. Medical therapy is not standardized and must be individualized. In patients who develop severe neurological symptoms such as obstructive hydrocephalus, surgery may be considered to avoid neurological sequelae. In our case, the execution of an ETV associated with medical therapy made it possible to obtain complete resolution of the clinical. A study of the endoscopic trajectory and the floor of the third ventricle could be performed to improve the surgical technique.
